# Comparison of cumulative dispersed energy between conventional phacoemulsification and femtosecond laser-assisted cataract surgery with two different lens fragmentation patterns

**DOI:** 10.1007/s10103-021-03321-1

**Published:** 2021-04-19

**Authors:** Hung-Yuan Lin, Shu-Ting Kao, Ya-Jung Chuang, Shuan Chen, Pi-Jung Lin

**Affiliations:** 1 Universal Eye Center, Zhong-Li, Taiwan; 2 Department of Optometry, Central Taiwan University of Science and Technology, Taichung, Taiwan; 3 Department of Ophthalmology, Fujian Medical University, Fujian Sheng, China; 4 Universal Eye Center, Ban-Qiao, Taiwan; 5 Universal Eye Center, Long-Tan, Taiwan; 6 Universal Eye Center, Taipei, Taiwan

**Keywords:** Cumulative dispersed energy, CDE, Cataract surgery, Femtosecond laser-assisted cataract surgery, FLACS, Lens fragmentation patterns, Grid pattern, Quadrant pattern, Conventional phacoemulsification

## Abstract

The purpose of the study is to compare the total ultrasound power used between eyes undergoing different lens fragmentation patterns of femtosecond laser-assisted cataract surgery (FLACS) and conventional phacoemulsification surgery (CPS). A total of 506 patient eyes underwent preoperative grading of lens opacity using the Lens Opacity Classification System III (LOCSIII). The eyes were divided into two subgroups: subgroup 1 had a LOCSIII grade of 1–3, and subgroup 2 had a LOCSIII grade of 4–6. The eyes underwent FLACS (LenSx) for clear corneal wound, capsulotomy, and lens fragmentation. Either a grid pattern or radial pattern was used for lens fragmentation. The eyes received one of the following three treatments: (1) CPS without femtosecond laser assistant, (2) FLACS with a grid pattern (FGP) lens fragment, or (3) FLACS with a quadrant pattern (FQP) lens fragment. The mean cumulative dispersed energy (CDE) for each subgroup and treatment was evaluated. The mean CDE was lower in the two FLACS groups (1.21±1.91 in FGP and 1.22±1.92 in FQP) than that in the CPG group (2.67±2.84). In subgroup 1, CDE was higher in the CPG group (1.54±1.18) as compared with the FLACS groups (0.16±0.31 in FGP and 0.74±1.17 in FQP; *P*<0.001). In subgroup 2, CDE was higher in the CPG (6.47±3.46) as compared with the FLACS groups (2.74±2.21 in FGP and 5.34±2.17 in FQP; *P<*0.001). CDE was lower in the two FLACS groups than that in the CPS group, and CDE was the lowest with FGP in both subgroups 1 and 2.

## Introduction

Cataract is the leading cause of blindness worldwide, but it can be resolved by cataract surgery using the phacoemulsification technique, which was developed in 1967 [1]. Although phacoemulsification instrumentation has evolved since the introduction of this technique, there are still many vision-threatening complications caused by phacoemulsification, such as corneal endothelial cell loss (ECL), vitreous loss, cystic macular edema, and postoperative infection [2, 3]. ECL was concluded on heat generated by ultrasonic tips and free radicals and fluid turbulence generated during ultrasonic energy delivery, which also accumulate with cumulative dispersed energy (CDE) [4–7]. To reduce those complications, the femtosecond laser was introduced in cataract surgery in 2009 to create corneal incisions, continuous curvilinear capsulorrhexis (CCC), and lens fragmentation [8]. Over the past decade, several studies have been conducted to evaluate the efficacy and safety of femtosecond laser-assisted cataract surgery (FLACS) versus conventional phacoemulsification surgery (CPS). FLACS was demonstrated to create more precise central CCC and a clear wound cut, which decrease refractive surprises such as myopic or hyperopic shift, unwanted surgically induced astigmatism, posterior chamber lens shift, increase in higher-order aberrations, and glare phenomena [9]. In addition, FLACS demonstrated a significant reduction in effective phacoemulsification time (EPT), ECL, and postoperative corneal edema, which is associated with improved visual outcomes and postoperative recovery [10, 11].

However, FLACS’s effect on CDE remains controversial. Although both Mastropasqua et al. and Hida et al. studied the LenSx platform, only Hida et al. found a lower CDE with FLACS as compared with CPS [12, 13]. In a large study by Osamah et al. of 1159 eyes, the authors found a moderate reduction in CDE with FLACS as compared with CPS [14]. Many factors contribute to the amount of CDE used with FLACS, such as nuclear opalescence and the different types of lens fragmentation patterns. There are two major lens fragmentation patterns in FLACS: the grid pattern and radial pattern. Tukezban et al. studied 71 eyes undergoing FLACS using different grid patterns and concluded that a smaller grid fragmentation is more beneficial with reduced EPT and ultrasound power [15]. Another study focused on FLACS using different lens fragmentation patterns, including three-plane chop and pie-cut pattern fragmentation; the results showed that the three-plane chop pattern had lower EPT, power, and CDE [16].

The aim of this study is to evaluate the effect of FLACS on CDE using two different lens fragment patterns as compared with that of CPS. We also collected patient profiles to examine the degree of nuclear sclerosis, with the aim of determining its influence on the average amount of CDE used. We hypothesize that the use of FLACS results in a lower amount of CDE than the use of CPS and that there is no difference in CDE using FLAC with a grid or radial lens fragmentation pattern as compared with that using CPS.

## Patients and methods

This retrospective case–control study included 506 consecutive eyes of patients undergoing either FLACS or CPS at a university eye center from January 2014 to June 2017 by the same experienced surgeon. Preoperative and postoperative patient data were recorded. All patients were fully informed of the risks and benefits of the procedure before undergoing the surgery, and they were given the option of FLACS. All patients signed an informed consent form before surgery. The cost of the surgery and intraocular lens was covered by the patient or their health insurance.

All patients included in the study had preoperative Lens Opacity Classification System III (LOCSIII) grade 1–6 nuclear sclerosis, diagnosed through slit-lamp examination by an experienced ophthalmologist. Patients with a history of prior ocular surgery, corneal scar, ocular inflammatory disease, end-stage glaucoma, zonular dehiscence, congenital cataract, and traumatic cataract were excluded. Patients initially included in the study were excluded if any severe intraoperative or postoperative complications occurred or in case of any missing clinical data.

**Fig. 1:**
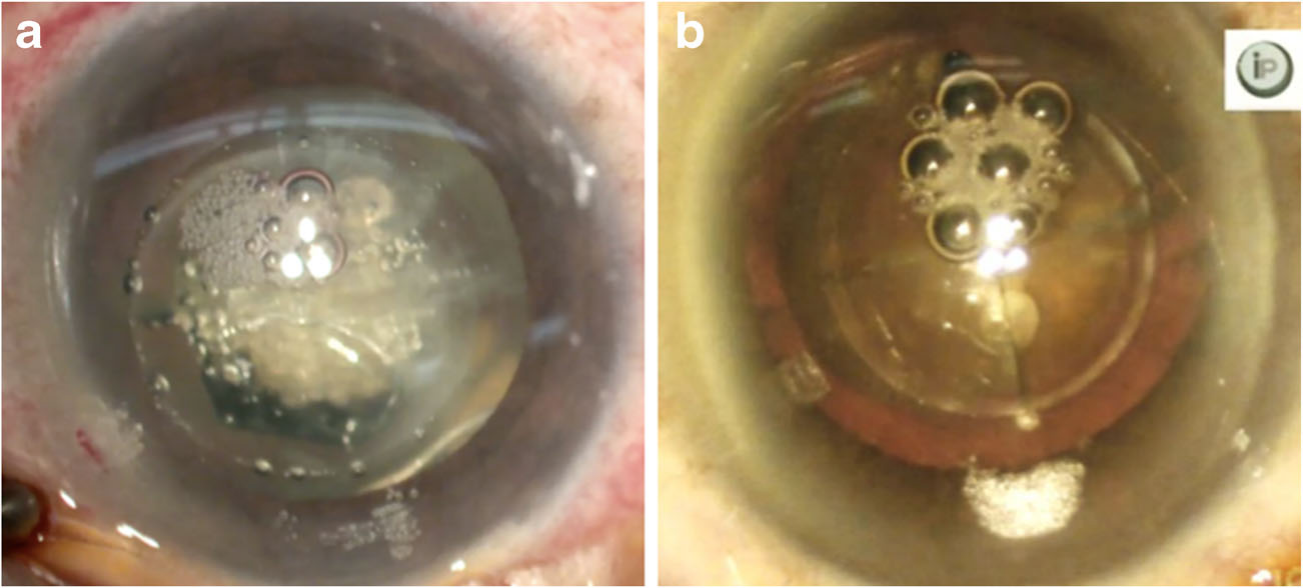
Lens fragmentation pattern in femtosecond laser-assisted cataract surgery. **a** Grid pattern and **b** radial (quadrant) pattern

Before the patients entered the protocol, they were allowed to select either CPS or FLACS. The eyes undergoing FLACS were randomly assigned to two lens fragmentations: a grid pattern or radial (quadrant) pattern (Fig. 1). The eyes were divided into three treatments as follows: (1) CPS without femtosecond laser assistance, (2) FLACS with a grid pattern (FGP) lens fragment, and (3) FLACS with a quadrant pattern (FQP) lens fragment. All patients were divided into two subgroups according to the degree of nuclear sclerosis, as follows: (1) subgroup 1, cataracts of LOCSIII grade 1–3; and (2) subgroup 2, cataracts of LOCSIII grade 4–6. Table 1 shows demographics of the patients included in the study.

**Table 1:** Patient demographics and surgical parameters of the eyes with CPS and with different lens fragmentation patterns in FLACS

Parameter	CPS Mean ± SD (%)	FGP Mean ± SD (%)	FQP Mean ± SD (%)	*P* value*
Eyes	288	113	105	
Subgroup 1 (%)	222 (77%)	67 (59%)	94 (90%)	
Subgroup 2 (%)	65 (23%)	46 (41%)	11 (10%)	
Age (years) Range	66.66 ± 8.99 37–92	64.64 ± 10.60 32–86	65.65 ± 9.27 43–87	0.142
Female sex (%)	56%	53%	62%	0.402
Mean CDE (%s)	2.67 ± 2.84	1.21 ± 1.91	1.22 ± 1.92	<0.001
Subgroup 1 (CDE)	1.54 ± 1.18	0.16 ± 0.31	0.74 ± 1.17	<0.001 <0.001#
Subgroup 2 (CDE)	6.47 ± 3.46	2.74 ± 2.21	5.34 ± 2.17	<0.001 0.001#

*CPS*, conventional phacoemulsification surgery; *FGP*, FLACS with grid pattern lens fragment; *FQP*, FLACS with quadrant pattern lens fragment; *FLACS*, femtosecond laser–assisted cataract surgery

Subgroup 1: LOCSIII grades 1–3, subgroup 2: LOCSIII grade 4–6

*Statistical analysis was performed using SPSS software version 16. For categorical variables (age and sex), chi-square test was used. One-way analysis of variance was used for comparison of the mean CDE among different types of surgical management

#Chi-square test was used only for the comparison of the mean CDE between the two FLACS treatments

All procedures were performed under topical anesthesia. Pretreatment of FLACS was performed with the patient in the supine position and using the LenSx (Alcon Laboratories Inc., Fort Worth, TX) platform and consisted of clear corneal incision (three-plane, 2.2-mm wide), capsulotomy (5.5-mm diameter), and lens fragmentation, consisting of either FGP (lens softened with a grid with a diameter of 560 mm) or FQP (lens segmented into quadrants of 560-mm diameter). After completion of laser pretreatment, the patient was transferred to the operation table, and phacoemulsification was performed. Patients undergoing CPS went directly to the operating room, and the chop technique was performed using the Centurion Vision System (Alcon Laboratories Inc.). All procedures were performed by the same surgeon. An intraocular lens was placed in the capsule bag in all cases, and all patients received the same postoperative treatment.

The exposure in this study was the type of cataract surgery: FLACS with one of two different types of lens fragmentations (i.e., FGP and FQP) or CPS. The outcome variable was the amount of intraoperative ultrasound energy, quantified by CDE, which was displayed on the screen of the Centurion Vision System at the end of the surgery. The other covariates were the degree of nuclear sclerosis, including a comparison between subgroup 1 and subgroup 2.

SPSS software version 16 was used for statistical analysis. For categorical variables such as age, we used a chi-square test. To compare CDE between different surgical management methods, one-way analysis of variance and chi-square test were used. Statistical significance was set as a *P* value <0.05 with a 95% confidence interval.

## Results

There was no significant difference in age (*P*=0.142) or gender (*P*=0.402) between the three groups. The mean CDE was significantly lower in the two FLACS groups (1.21±1.91 in FGP and 1.22±1.92 in the FQP group) than that in the CPS group (2.67±2.84). We also classified cataracts of all patients into two subgroups based on the degree of nuclear sclerosis. In subgroup 1, CDE was significantly higher in the CPS group (1.54±1.18) as compared with that in the FLACS groups (0.16±0.31 in FGP and 0.74±1.17 in FQP; *P*<0.001). In subgroup 2, CDE was significantly higher in the CPS group (6.47±3.46) as compared with that in the FLACS groups (2.74±2.21 in the FGP group and 5.34±2.17 in the FQP group; *P*<0.001). We also compared the two FLACS groups using a chi-square test to determine which group had the lowest CDE. We found a significant difference (*P*<0.001) in CDE between the two subgroups. In conclusion, CDE was lower in the two FLACS groups as compared with that in the CPS group, and CDE was lowest in the FGP subgroup among all cataract groups.

## Discussion

The femtosecond laser can focus at a specific depth within the cornea or lenticular tissue, as the infrared wavelengths emitted are not absorbed by the nearby tissue. This precise focus was applied in FLACS to complete the cataract surgical procedure manually [17]. FLACS has been reported to offer potential advantages of reduced surgical complications and better visual outcomes with more surgical precision and reproducibility [18]. However, FLACS is also associated with the problems of increased length of surgery and higher surgical costs [19]. Over the past decades, comparisons between FLACS and CPS have been discussed and continue to be controversial.

FLACS has demonstrated some benefits, including increased circular CCC, which reduces the decentration of the intraocular lens and the incidence of posterior capsular opacity [20, 21]. However, studies have reported an increase in the incidence of anterior capsule tear in FLACS, mainly as a result of the irregularity of the capsule edge [22, 23]. FLACS has also been applied to lens fragmentation and was shown to reduce ultrasound energy power, CDE, and EPT [13, 23–25]. The use of excessive ultrasound power during surgery can increase the turbulence of the anterior chamber, cavitation bubbles, heat, and oxidative radical generation, which result in endothelial cell injury and prolonged surgical recovery [26, 27]. The lens should be pretreated with the femtosecond laser to reduce thermal or ultrasound energy during the procedure. The importance of reduced ultrasound energy use is well established in the literature, as it decreases corneal edema and ECL [10, 28].

In a previous randomized clinical trial (RCT) of 76 eyes that compared the LenSx with CPS, the authors reported a reduction of ultrasound energy but no difference in EPT [29]. Chen et al. published a large meta-analysis that comprised 989 eyes from nine RCTs and concluded that FLACS significantly reduced the mean ultrasound energy and EPT as compared with CPS. The central corneal thickness was significantly lower in FLACS at 1 day of follow-up, but the central corneal thickness and corneal endothelial cell count were comparable at 1 week of follow-up or longer. FLACS achieved a better visual outcome at 1 week and 6 months after the operation, but the difference was not significant 1–3 months postoperation [25]. Another meta-analysis comparing FLACS and CPS published by Popovic et al. collected 14,567 eyes from 15 RCTs and 22 conservative cohort studies. They found that FLACS has benefits over EPT, including capsulotomy circularity, and ECL, but there was no difference in visual or refractive outcomes [30]. Hida et al. published an RCT of 400 patients (200 with FLACS and 200 with CPS), who received treatment with an active fluidics phacoemulsification machine. They found a reduction in CDE in the FLACS group, but this did not translate into a difference in ECL [13]. The total ultrasound power used during the surgery was calculated by the value of CDE, which revealed a significant reduction in power in the FLACS group as compared with that in the CPS group [15]. However, the effect of FLACS on ECL reduction is still controversial. The ECL is related to the grade of nuclear opacity, increased CDE, prolonged aspiration time, and increased amount of fluid used during the surgical procedure [31]. We hypothesized that there might be a threshold of CDE to cause ECL, which might be individually different. The reduction of CDE in FLACS must be large enough to be below the threshold, which could influence the outcome of EPL. This might explain the significant reduction in CDE but the lack of difference in ECL in FLACS.

The reduced CDE in FLACS might be influenced by the pattern of lens fragmentation and the degree of nuclear sclerosis. As the nuclear hardness and density increase, more EPT and CDE are required during surgery and the length of postoperative visual rehabilitation is prolonged. A hard nucleus with FLACS leads to a reduction in corneal endothelial damage, anterior and posterior capsule rupture, and postoperative corneal edema [10, 32, 33]. In our study, we hypothesized that the reduction of CDE might be proportional to the density of the lens nucleus. In addition to the cataract density, the lens fragmentation pattern also influences the ultrasound power. Harvey et al. reported that extensive fragmentation, such as a pie-cut pattern, appears to lower the EPT and CDE and might potentially reduce surgical complications as compared with the three-plane chop pattern [16]. The grid pattern of lens fragmentation in FLACS was determined to reduce EPT and ultrasound power as compared with the quadrant pattern [15]. However, no study has demonstrated the different patterns of lens fragmentation in FLACS by performing subgroup analysis based on nuclear density grading.

In our study, we reviewed patient history and classified their eyes into two subgroups based on the density of the lens nucleus. All patients received cataract surgery with different lens fragmentations, including CPS without femtosecond laser assistance, FLACS with FGP, and FLACS with FQP. We analyzed the value of CDE in the different groups and reached the following conclusions. First, CDE was significantly lower in the two FLACS groups as compared with that in the CPS group, which is consistent with the findings of previous studies. In subgroup 1 (cataract grading 1–3 of LOCSIII), FLACS with FGP significantly decreased the amount of CDE to 1.458 (%-s) and 1.45 (%-s) in FLACS with FQP group compared with the CPS group. In subgroup 2 (cataract grading 4–6 of LOCSIII), FLACS with FGP significantly decreased the amount of CDE to 3.726 (%-s) and to 1.139 (%-s) in FLACS with FQP. FLACS with FGP was demonstrated to have the lowest CDE in both subgroups (0.16±0.31 in subgroup 1 and 2.75±2.21 in subgroup 2). Theoretically, more extensive and deeper fragmentation would lead to a greater reduction in ultrasound power for nuclear destruction. According to our results, FLACS could reduce CDE in both lens fragmentation patterns as compared with CPS, and FLACS with FGP resulted in the lowest CDE of all eyes. In the eyes with mild to moderate nuclear density, there was little difference in CDE in the FGP versus FQP groups with femtosecond laser assistance, which might result in no significant difference in postoperative clinical complications, such as corneal edema or ECL. However, in a hard nucleus, FLACS with FGP might result in less ECL, less postoperative corneal edema, and fast rehabilitation time. We suggest that FLACS with FGP could be performed in patients with dense cataract.

One of the limitations of this study is the absence of randomization, and this retrospective case–control study is less reliable than RCTs. Because of the extra payment required for FLACS, patients had the right to choose their desired surgical procedure. Additional limitations include the small sample size and the fact that we had a single, experienced surgeon perform the same phacoemulsification techniques with the same machine, which might be different from another surgeon using different phacoemulsification techniques and machine. Further studies including more surgeons and different techniques are expected. More phacoemulsification and FLACS parameters such as EPT, docking time, irrigation fluid, and postoperative visual and refractive outcomes should be collected for further analysis. Finally, long-term follow-up of endothelial cell count loss should be studied.

In conclusion, as compared with CPS, FLACS with two types of lens fragmentation demonstrated reduced CDE in patients with all types of cataract. We suggest using FLACS with FGP to complete the surgeries of eyes with a hard nucleus, as this procedure might result in a better prognosis.
